# *Coryneumheveanum* sp. nov. (Coryneaceae, Diaporthales) on twigs of Para rubber in Thailand

**DOI:** 10.3897/mycokeys.43.29365

**Published:** 2018-12-06

**Authors:** Chanokned Senwanna, Kevin D. Hyde, Rungtiwa Phookamsak, E. B. Gareth Jones, Ratchadawan Cheewangkoon

**Affiliations:** 1 Department of Entomology and Plant Pathology, Faculty of Agriculture, Chiang Mai University, Chiang Mai 50200, Thailand; 2 Center of Excellence in Fungal Research, Mae Fah Luang University, Chiang Rai 57100, Thailand; 3 Key Laboratory for Plant Diversity and Biogeography of East Asia, Kunming Institute of Botany, Chinese Academy of Sciences, Kunming 650201, Yunnan, People’s Republic of China; 4 World Agroforestry Centre, East and Central Asia, Heilongtan, Kunming 650201, Yunnan, People’s Republic of China; 5 Department of Biology, Faculty of Science, Chiang Mai University, Chiang Mai 50200, Thailand

**Keywords:** 1 new species, Ascomycota, *
Hevea brasiliensis
*, Phylogeny, Taxonomy

## Abstract

During studies of microfungi on para rubber in Thailand, we collected a new *Coryneum* species on twigs which we introduce herein as *C.heveanum* with support from phylogenetic analyses of LSU, ITS and TEF1 sequence data and morphological characters. *Coryneumheveanum* is distinct from other known taxa by its conidial measurements, number of pseudosepta and lack of a hyaline tip to the apical cell.

## Introduction

The para rubber tree (*Heveabrasiliensis*) is a tropical plant belonging to family Euphorbiaceae (Priyadarshan et al. 2009). Para rubber tree is the major commercial source of natural rubber, which is used in all kinds of manufactured products, including tyres, medical appliances and agricultural equipment, in addition, rubber-wood is used in the furniture industry (Priyadarshan et al. 2009, Rippel and Galembeck 2009, Häuser et al. 2015, Herrmann et al. 2016). The total para rubber tree plantation area in South East Asia exceeds more than 5 million hectares (Vongkhamheng et al. 2016). In Thailand, para rubber tree plantation area covers more than 3 million hectares and this number has increased every year (Kromkratoke and Suwanmaneepong 2017, Romyen et al. 2018). This important perennial crop is currently often affected by plant pathogenic fungi which can substantially decrease the quality and quantity of rubber yield (Liu et al. 2018). Many taxa have proven to be serious pathogens worldwide, causing severe leaf spot formation, defoliation, shoot die-back and stem cankers (Jayasinghe 2000, Nyaka Ngobisa et al. 2015, Trakunyingcharoen et al. 2015, Liyanage et al. 2016, Liu et al. 2018). Nonetheless, information about the diversity of phytopathogenic taxa on para rubber from Thailand is generally lacking and currently there are only thirteen reports (Farr and Rossman 2018). Thus, the main objective of our project is to survey and study the diversity of microfungi associated with para rubber trees in Thailand. During the survey, we found a *Coryneum* species associated with canker disease on twigs of para rubber. This work is based on a combination of morphology and molecular data for identification this taxon.

Many *Coryneum* species have been reported as phytopathogens causing tree canker (Strouts 1972, Gadgil and Dick 2007, Horst 2013, Senanayake et al. 2017). This genus was introduced by Nees von Esenbeck (1817) to accommodate *C.umbonatum* as the type species. Historically, *Coryneum* species have relied on morphological studies and only a few species are supported by sequence data in GenBank. Many species causing tree canker, previously known as *Coryneum* were transferred to other genera e.g. *Seiridium*, *Seimatosporium* and *Wilsonomyces* (Sutton 1980, Raddi and Panconesi 1981, Marin-Felix et al. 2017). Recently, research has clarified the taxonomic position of the family Coryneaceae based on morphological and molecular data (Rossman et al., 2015; Senanayake et al., 2017; Wijayawardene et al., 2018). Currently 123 *Coryneum* species are listed in Index Fungorum (2018). Molecular analyses, using sequence data of LSU, ITS and TEF1 regions, has supplemented traditional taxonomic methods, enabling a more precise and rapid identification of species in the genus *Coryneum* (Senanayake et al. 2017, 2018, Fan et al. 2018). The correct identification of pathogenic fungi is necessary to implement appropriate quarantine decisions, suitable control strategies and to promote an understanding of the evolution of new pathogens and the movement of fungi between continents.

## Material and methods

### Collections, morphological studies and isolation

Fresh materials were collected from Chiang Rai, Thailand in 2016. Specimens were taken to the laboratory in zip lock bags and observed with a Motic SMZ 168 series stereomicroscope and photographed with an Axio camera on a Zeiss Discover V8 stereomicroscope. Sections of the conidiomata were mounted in double-distilled water (ddH_2_O) for morphological structures and photography. Images were taken using a Canon 600D camera on a Nikon ECLIPSE 80i microscope. All measurements were calculated using Tarosoft® Image Framework programme v.0.9.0.7. Photoplates were made using Adobe Photoshop CS6 version 13.0 (Adobe Systems U.S.A.). The specimens were deposited in the Mae Fah Luang University Herbarium, Chiang Rai, Thailand (MFLU). Living cultures were deposited in Mae Fah Luang University Culture Collection (MFLUCC) in Thailand and duplicated at the Kunming Culture Collection (KUMCC). Faces of Fungi and Index Fungorum numbers are registered as described in Jayasiri et al. (2015) and Index Fungorum (2018).

### DNA extraction, PCR and DNA sequencing

Genomic DNA was extracted from mycelium using Biospin Fungus Genomic DNA Extraction Kit (BioFlux®, Hangzhou, P.R. China) following the manufacturer’s protocol. The DNA product was kept at 4 °C for the DNA amplification and maintained at -20 °C for long term storage. The DNA amplification was carried out by polymerase chain reaction (PCR) using three genes, the 28S large subunit (LSU), internal transcribed spacer (ITS) and translation elongation factor 1 alpha gene (TEF1). The LSU gene was amplified by using the primers LROR and LR5 (Vilgalys and Hester 1990), the ITS gene was amplified by using the primers ITS5 and ITS4 (White et al. 1990) and the TEF1 gene was amplified using the primers EF1-728F (Carbone and Kohn 1999) and EF2 (O’Donnell 1998). The amplification reactions were performed in 25 μl final volumes contained of 8.5 μl of sterilized ddH_2_O, 12.5 μl of 2 × Easy Taq PCR Super Mix (mixture of Easy Taq TM DNA Polymerase, dNTPs and optimised buffer (Beijing Trans Gen Biotech Co., Chaoyang District, Beijing, PR China), 1 μl of each forward and reverse primers (10 pM) and 2 μl of DNA template. The PCR thermal cycle programme for LSU and ITS gene amplification was provided as initially 94 °C for 3 mins, followed by 35 cycles of denaturation at 94 °C for 1 min, annealing at 55 °C for 50 secs, elongation at 72 °C for 1 min and final extension at 72 °C for 10 mins. The PCR thermal cycle programme for TEF1 gene amplification was provided as initially 94 °C for 5 mins, followed by 40 cycles of denaturation at 94 °C for 45 secs, annealing at 52 °C for 30 secs, elongation at 72 °C for 1.30 mins and final extension at 72 °C for 6 mins. PCR products were sequenced by Sangon Biotech Co., Shanghai, China. Nucleotide sequences were deposited in GenBank (Table 1).

### Phylogenetic analysis

Phylogenetic analyses were conducted based on a combined gene of LSU, ITS and TEF1 sequence data. Sequence data of Coryneaceae from previous studies and representative strains of major classes in Diaporthales were downloaded from GenBank to supplement the dataset (Table 1). The combined dataset consisted of 45 sequences including our newly generated sequences. *Phaeoacremoniumaleophilum* (CBS 63194) and *P.vibratile* (CBS 117115) were selected as the outgroup taxa. The combined LSU, ITS and TEF1 gene dataset were initially aligned by using MAFFT version 7 (Katoh et al. 2017; http://mafft.cbrc.jp/alignment/server/) and improved manually, where necessary, in BioEdit v.7.0.9.1 (Hall 1999) and MEGA7 (Kumar et al. 2015). The final alignment of the combined LSU, ITS and TEF1 sequence datasets was analysed and inferred the phylogenetic tree based on maximum likelihood (ML), maximum parsimony (MP) and Bayesian inference analyses (BI).

**Table 1.** Isolates utilized in the phylogenetic tree and their GenBank and culture accession numbers.

Taxa	Culture AC no.	GenBank Accession number		
		ITS	LSU	TEF1
* Asterosporium asterospermum *	KT2125	_	AB553743	_
* Asterosporium asterospermum *	KT2138	_	AB553744	_
* Chaetoconis polygoni *	CBS 405.95	_	EU754141	_
* Coryneum castaneicola *	43-1	_	MH683551	_
* Coryneum castaneicola *	43-2	MH683560	MH683552	_
* Coryneum depressum *	AR 3897	_	EU683074	_
*** Coryneum heveanum ***	**MFLUCC 17-0369**	**MH778707**	**MH778703**	**MH780881**
*** Coryneum heveanum ***	**MFLUCC 17-0376**	**MH778708**	**MH778704**	**_**
* Coryneum modonia *	AR 3558	_	EU683073	_
* Coryneum perniciosum *	CBS 130.25	MH854812	MH866313	_
* Coryneum umbonata *	CBS 199.68	MH859114	MH870828	_
* Coryneum umbonatum *	AR 3541*	_	EU683072	_
* Coryneum umbonatum *	MFLUCC 13-0658*	MF190120	MF190066	MF377574
* Coryneum umbonatum *	MFLUCC 15-1110*	MF190121	MF190067	MF377575
* Crinitospora pulchra *	CBS 138014	KJ710466	KJ710443	_
* Cytospora centravillosa *	MFLUCC 17-1660	MF190122	MF190068	_
* Cytospora centravillosa *	MFLU 17-0887	MF190123	MF190069	_
* Cytospora melanodiscus *	Jimslanding2	JX438621	_	JX438605
* Cytospora translucens *	CZ320	FJ755269	FJ755269	_
* Diaporthe azadirachtae *	TN 01	KC631323	_	_
* Diaporthe eres *	AR 5193*	KJ210529	_	KJ210550
* Diaporthe eres *	MFLUCC 17-1668	MF190138	MF190081	MF377595
* Diaporthe maytenicola *	CBS 136441	KF777157	KF777210	_
* Hyaliappendispora galii *	MFLUCC 16-1208	MF190150	MF190095	MF377587
* Lamproconium desmazieri *	MFLUCC 15-0870*	KX430134	KX430135	MF377591
* Lamproconium desmazieri *	MFLUCC 15-0872	KX430138	KX430139	MF377593
* Macrohilum eucalypti *	CPC 10945*	DQ195781	DQ195793	_
* Macrohilum eucalypti *	CPC 19421*	KR873244	KR873275	_
* Pachytrype princeps *	Rogers s.n.*	_	FJ532382	_
* Pachytrype rimosa *	FF1066	_	FJ532381	_
* Phaeoacremonium aleophilum *	CBS 631.94	AF266647	AB278175	KF764643
* Phaeoacremonium vibratile *	CBS 117115	KF764573	DQ649065	KF764645
* Phaeoappendispora thailandensis *	MFLUCC 13-0161*	MF190157	MF190102	_
* Phaeoappendispora thailandensis *	MFLU 12-2131	MF190158	MF190103	_
* Phaeodiaporthe appendiculata *	CBS 123821*	KF570156	KF570156	_
* Prosopidicola mexicana *	CBS 113529*	AY720709	KX228354	_
* Prosopidicola mexicana *	CBS 113530*	AY720710	_	_
* Rossmania ukurunduensis *	AR 3484*	_	EU683075	_
* Stegonsporium acerophilum *	CBS 117025	EU039982	EU039993	EU040027
* Stegonsporium pyriforme *	CBS 117023	EU039971	EU039987	EU040001
* Stilbospora ellipsosporum *	WJ 1840	_	AY616229	_
* Stilbospora macrosperma *	CBS 121883*	JX517290	JX517299	_
* Sydowiella depressula *	CBS 813.79	_	EU683077	_
* Sydowiella fenestrans *	CBS 125530*	JF681956	EU683078	_
* Valsella salicis *	AR 3514	_	EU255210	EU222018

Note: AR: AR, Amy Rossman; ATCC: American Type Culture Collection, Virginia, USA; BCRC, Bioresource Collection and Research Center, Taiwan; CBS: Westerdijk Fungal Biodiversity Institute, Utrecht, Netherlands; CFCC: China Forestry Culture Collection Center, Beijing, China; CPC: Culture Collection of Pedro Crous, Netherlands; FF: F.A. Fernández; KT: K. Tanaka; MFLU: MAFF: MAFF Genebank, Ministry of Agriculture Forestry and Fisheries, USA; Mae Fah Luang University Herbarium, Chiang Rai, Thailand; MFLUCC: Mae Fah Luang University Culture Collection, Chiang Rai, Thailand; WJ: W. Jaklitsch. The newly generated sequences are indicated in bold. The strains from generic type species are marked by an asterisk (*).

The estimated evolutionary model of Bayesian inference and maximum likelihood were performed independently for each locus using MrModeltest v. 2.3 (Nylander 2004) implemented in PAUP v. 4.0b10 (Swofford 2002). The best-fit model resulted as GTR+I+G model for each locus under the Akaike Information Criterion (AIC).

Maximum likelihood analysis was performed by Randomized Accelerated Maximum Likelihood (RAxML) (Stamatakis 2008) version 7.4.2 (released by Alexandros Stamatakis on November 2012) implemented in raxmlGUI v.1.0 (Stamatakis et al. 2008, Silvestro and Michalak 2011). The search strategy was set to rapid bootstrapping at 1,000 replicates.

Maximum parsimony analysis was performed using PAUP v 4.0b10 (Swofford 2002). Trees were inferred using the heuristic search function with 1,000 random stepwise addition replicates and tree bisection-reconnection (TBR) as the branch-swapping algorithm. All informative characters were unordered and of equal weight. The consistency index (CI), retention index (RI), rescaled consistency index (RC) and homoplasy index (HI) were calculated. Statistical supports for branches of the most parsimonious tree were estimated using maximum parsimony bootstrap (BS) analysis with 1,000 bootstrap replicates.

Bayesian inference was performed in MrBayes v. 3.2.2 (Ronquist and Huelsenbeck 2003) with the best-fit model of sequences evolution under the Akaike Information Criterion (AIC). Bayesian posterior probabilities (BY) (Rannala and Yang 1996, Zhaxybayeva and Gogarten 2002) were determined by Markov Chain Monte Carlo Sampling (BMCMC). Six simultaneous Markov chains were run from random trees for one million generations and trees were sampled every 100^th^ generation. The first 20% of generated trees representing the burn-in phase of the analysis were discarded and the remaining trees were used for calculating posterior probabilities (BY) in the majority rule consensus tree.

The phylogenetic tree was shown in FigTree V.1.4.3 (Rambaut 2016) and drawn and converted to tiff file in Microsoft PowerPoint 2013 and Adobe Photoshop CS6 version 13.0 (Adobe Systems U.S.A.). The final alignment and tree were deposited in TreeBASE (http://www.treebase.org/) under the submission ID 23550.

## Results

### Phylogenetic analysis

**Figure 1.**
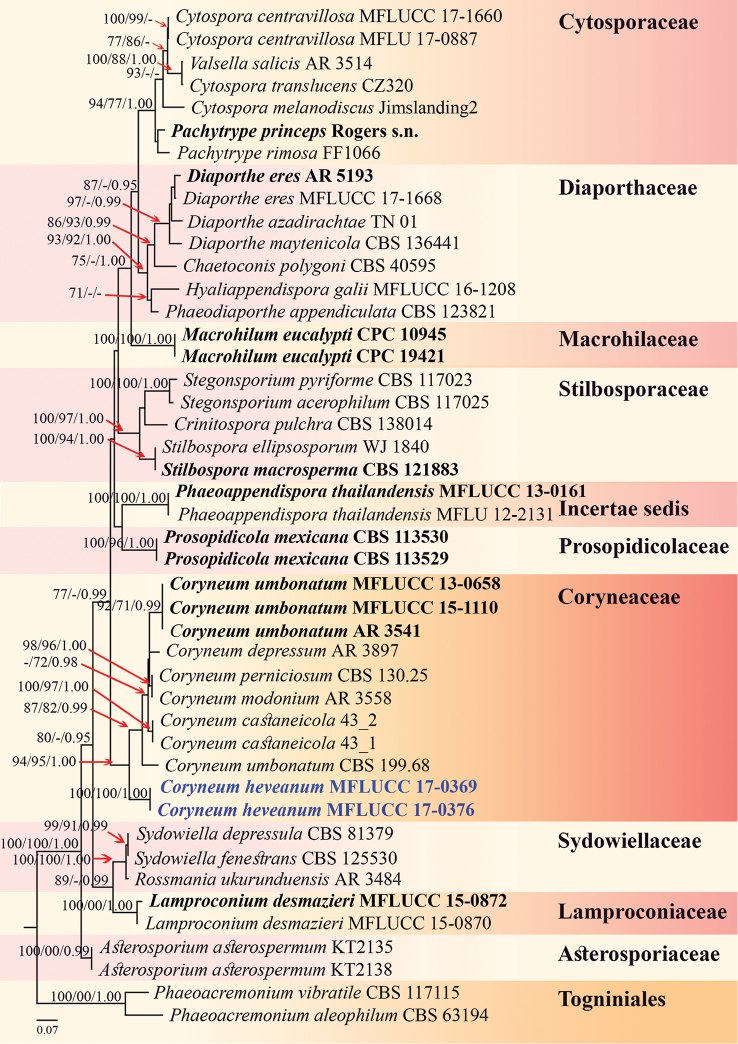
Maximum likelihood (RAxML) based on analysis of a combined dataset of LSU, ITS and TEF1 sequence data representing Diaporthales. Bootstrap support values for maximum likelihood (ML, left), maximum parsimony (MP, middle) greater than 70% and Bayesian posterior probabilities (BY, right) equal to or greater than 0.95 are indicated at the nodes. The tree is rooted to *Phaeoacremoniumaleophilum* (CBS 63194) and *P.vibratile* (CBS 117115). The newly generated sequences are in blue. The strains from generic type species are in black bold.

The dataset consisted of 45 taxa including the new taxa (Figure 1). The combined LSU, ITS and TEF1 sequence data including 2040 total characters, were analysed based on Bayesian inference, maximum likelihood and maximum parsimony analysis. RAxML analysis of the combined dataset had 996 distinct alignment patterns and 39.23% of undetermined characters or gaps. Maximum parsimony had 1191 constant characters, 151 variable parsimony-uninformative characters and 690 parsimony-informative characters. The most parsimonious tree is shown where TL = 2336, CI = 0.607, RI = 0.716, RC = 0.435, HI = 0.393. Bayesian posterior probabilities (BY) from MCMC were evaluated with the final average standard deviation of split frequencies = 0.005452. Phylogenetic analysis from ML, MP and BI gave trees with similar overall topologies of the generic placement and in agreement with previous studies (Senanayake et al. 2017, Fan et al. 2018, Yang et al. 2018). The final RAxML tree of the combined dataset is shown in Figure 1, with a final ML optimisation likelihood value of -13004.6966291. The phylogeny shows that *Coryneumheveanum* forms a distinct lineage in *Coryneum* with strong support (94% ML, 95%MP and 1.00 BY) and in a sister clade to *C.umbonatum*, *C.depressum*, *C.modonium*, *C.perniciosum* and *C.castaneicola*.

## Taxonomy

### Coryneum heveanum

Taxon classificationFungiDiaporthalesPseudovalsaceae

Senwanna, Cheewangkoon & K.D. Hyde sp. nov.

Index Fungorum number: IF555338

Facesoffungi number: FoF 04873

Figure 2

#### Etymology.

Named after the host on which it occurs, *Heveabrasiliensis*.

#### Type.

THAILAND, Chiang Rai Province, Wiang Chiang Rung District, on twigs (attached on tree) of *Heveabrasiliensis*, 1 November 2016, C. Senwanna, RBCR003 (MFLU 18-0936, holotype), ex-type living culture MFLUCC 17-0369, KUMCC 18-0106; Dry culture from ex-type MFLU 18-0936); *ibid*., RBCR016 (MFLU 17-1982, living culture MFLUCC 17-0376, dry culture MFLU 18-0937, MFLU 18-0938)

#### Description.

*Associated with canker* on twigs of *Heveabrasiliensis*. Asexual morph: *Conidiomata* acervular, solitary, erumpent through the outer periderm layers of host, scattered, surface tissues above slightly dome-shaped, black, velvety, formed of brown cell, thick-walled *textura angularis*, 145–540 µm diam. *Conidiophores* short, cylindrical, apically pale brown, paler at the base, smooth, septate, branched at the base, arising from basal stroma, 22–37 × 4–8 μm (*x*‒ = 28.5 × 5.6 μm, n = 15). *Conidiogenous cell* annellidic, integrated, terminal, cylindrical, medium brown, truncate apex, with 1-3 slightly percurrent proliferations, 6–17 µm long (*x*‒ = 10.7 μm, n = 20). *Conidia* curved, clavate to fusiform, dark brown, smooth-walled, 4–6-pseudo-septa, sometimes with apical and basal cells darker than other cells, rounded or sometime truncate at apex, truncate and black at the base, (40–)43–53(–68) × (14–)15–20 μm (*x*‒ = 48.7 × 17.3 μm, n = 85). *Appressoria* hyaline, globose to sub globose, thick-walled, 4–11 μm wide (*x*‒ = 7.1 μm, n = 20).

#### Cultural characteristics.

**Figure 2.**
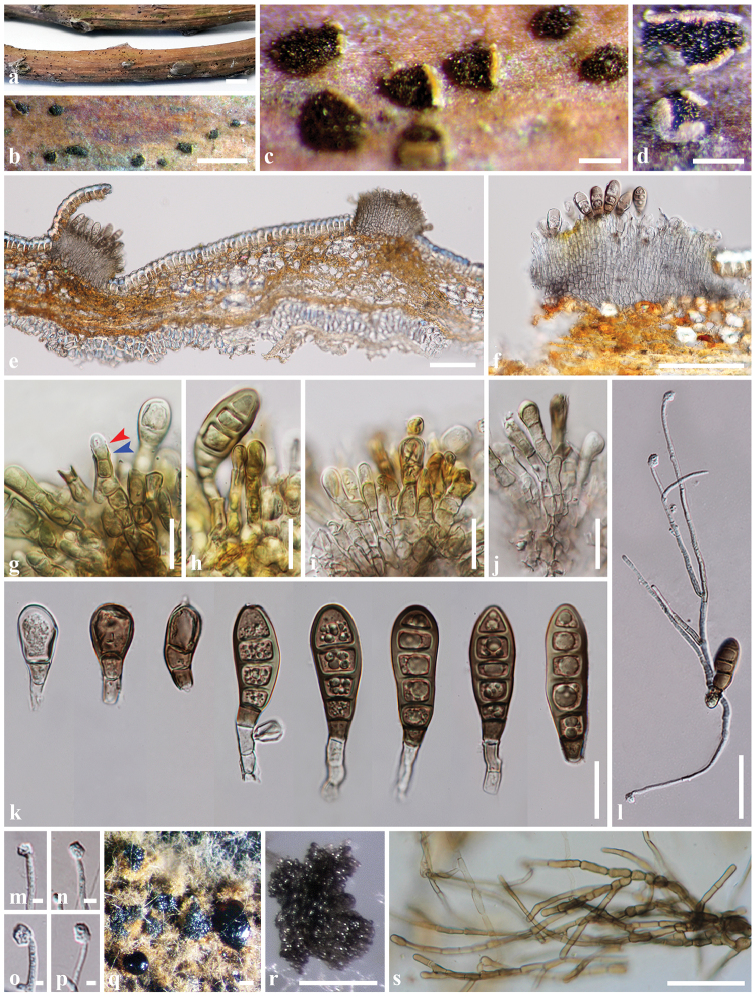
*Coryneumheveanum* (MFLU 18-0936). **a−d** Conidiomata on host surface **e−f** Acervuli **g** Conidiogenesis (annellidic; red arrow, proliferation; blue arrow) **h−j** Conidiophores, conidiogeneous cells with conidia **k** Conidia **l** Germinated spores **m−p** Appressoria **q−r** Mass of conidia on PDA after 6 months **s** Mycelium on PDA after 6 months. Scale bars: 5 mm (**a**), 1000 µm (**b**), 200 µm (**c, d, r**), 100 µm (**e, f, q**), 20 µm (**g−k**), 50 µm (**l, s**), 5 µm (**m−p**).

Conidia germinated on MEA within 24 h with germ tubes produced from one or both end cells, mostly from basal cell of conidia. Colonies on MEA reaching 20–25 mm diam. after 4 weeks at 25–30 °C, colonies circular, medium dense, cottony, margin wavy, superficial, slightly effuse, radially striated; colony from above, white, edges with more aerial mycelium than centre in the beginning and later become white grey, smooth with edge entire; from below: white to cream at the margin, yellowish-green in the centre in the beginning and later become dark green; not producing pigmentation in agar. Colonies on PDA reaching 10–15 mm diam. after 4 weeks at 25–30 °C, colonies circular, medium dense, cottony, slightly effuse, dark green with brown aerial mycelium on surface; not producing pigmentation in agar. Conidial masses were observed in PDA culture after 6 months at 25–30 °C. Mass of conidia dark brown to black, extruding on colony or tip of mycelium (Figure 2 q, r). Mycelium superficial and immersed, dark brown, hyphae branched, septate, constricted at septa, thick, smooth-walled (Figure 2 s).

#### Additional GenBank number.

SSU (primer NS1 and NS4; White et al. 1990) MH778705; MFLUCC 17-0369, MH778706; MFLUCC 17-0376, TEF1 (primer EF1-983F and EF1-2218R; Rehner 2001) MH780882; MFLUCC 17-0376.

#### Notes.

Phylogenetically, *Coryneumheveanum* clustered in the same clade with *C.umbonatum*, *C.depressum*, *C.modonium*, *C.perniciosum* and *C.castaneicola* with high statistical support. Based on morphological characters, the conidia of *C.castaneicola*, *C.depressum*, *C.elevatum*, *C.modonium* and *C.umbonatum* have slightly curved conidia with an apical cell with a hyaline tip, while *C.heveanum*, *C.castaneicola* and *C.perniciosum* lack a hyaline tip (Table 2) (Briosi and Farneti 1908, Sutton 1980, Gadgil and Dick 2007, Senanayake et al. 2017, 2018). *Coryneumheveanum* is similar to *C.betulinum*, *C.perniciosum*, *C.psidi* and *C.pyricola* in having broadly fusiform or clavate conidia but differs in size of conidia and number of pseudosepta (Table 2).

## Discussion

Fungi on para rubber (*Heveabrasiliensis*) can be pathogens, saprobes or endophytes (Rocha et al. 2011, Seephueak et al. 2011, Ghazali 2013, Nyaka Ngobisa et al. 2015, Hyde et al. 2018, Senwanna 2017, 2018). Fungal endophytes on para rubber have been comparatively well-studied (Gasiz and Chaverri 2010, Rocha et al. 2011, Déon et al. 2012, Martin et al. 2015), while few studies have investigated saprobic fungi or fungi associated with para rubber (Cai et al. 2013, Trakunyingcharoen et al. 2015). However, previous studies reporting saprobic taxa based on morphology, are available (Seephueak et al. 2011, Seephueak 2012). In this study, we introduced a new species, *Coryneumheveanum*, found on twigs of para rubber, based on morphological characters and phylogenetic analyses.

**Table 2.** Synopsis of recorded *Coryneum* species (asexual morph) (Related to this research).

**Taxa**	**Size (µm)**			**Host records**
	**Conidiomata**	**Conidiophores**	**Conidia; Number of pseudo-septate**	
*Coryneumbetulinum* (Sutton 1980)	–	–	31–36 × 14–17; 4–5	*Betularubrum* (Betulaceae)
*C.castaneicola* (Sutton 1980)	–	–	57–80 × 10–13; apical cell with a hyaline tip; 6–7	*Castaneadentata* (Fagaceae)
*C.depressum* (Sutton 1980)	–	–	44–53 × 19–23; apical cell with a hyaline tip; 4–5(–6)	*Quercus* spp. (Fagaceae)
*C.elevatum* (Sutton 1980)			56–70 × 24–32; apical cell with a hyaline tip; 6–7	*Quercus* spp. (Fagaceae)
*C.heveanum* This study	145–540	22–37 × 4–8	(40–)43–53(–68) × (14–)15–20; 4–6	*Heveabrasiliensis* (Euphorbiaceae)
*C.modonium* (Sutton 1980)	–	–	50–71 × 14–19; apical cell with a hyaline tip; 5–8	*Castanea* spp. (Fagaceae)
*C.perniciosum* (Briosi and Farneti 1908)	–	–	40–50 × 13–15; 5–7	*Castanea* sp. (Fagaceae)
*C.psidi* (Sutton 1980)	–	–	25–40 × 14–17; 5–6	*Psidiumguajava* (Myrtaceae)
*C.pyricola* (Sutton 1980)	–	–	61–70 × 24–32; 5–7	*Pyrus* sp. (Rosaceae)
*C.umbonatum* (*Pseudovalsalongipes*) (Wehmeyer 1926)	–	–	47–104 × 10–14; 3–8	*Quercuscoccinea* (Fagaceae)
*C.umbonatum* (Gadgil and Dick 2007, Sutton 1980)	1500–2200	(10–) 27.5–47	57–72 × 14–16; apical cell with a hyaline tip; 5–7	*Quercus* spp. (Fagaceae), *Castaneasativa* (Fagaceae)
*C.umbonatum* (Senanayake et al. 2017)	1000–1300 × 500–550	20–35 × 4–7	42–56 × 13–16; apical cell with a hyaline tip; 4–6	*Quercus* sp. (Fagaceae)
*C.umbonatum* (Senanayake et al. 2018)	450 × 700	20–30 × 3–6	35–45 × 8–10; apical cell with a hyaline tip; 4–6	*Quercuspetraea* (Fagaceae)

*Coryneum* species are phytopathogenic fungi associated with twig blight, canker and dieback disease with some species reported as saprobes (Carter 1914, Strouts 1972, Gadgil and Dick 2007, Senanayake et al. 2018). Host-specificity of *Coryneum* has not yet been clarified and species have been recorded from various plant families worldwide (i.e. Betulaceae, Clusiaceae, Cupressaceae, Fagaceae, Hippocastanoideae, Malvaceae, Myrtaceae, Rosaceae, Ulmaceae) (Wehmeyer 1926, Strouts 1972, Sutton 1980, Senanayake et al. 2017, 2018, Farr and Rossman 2018). Until recently, these taxa have primarily been identified by their morphology i.e. Sutton (1980) and only a few species are supported by molecular data with nine sequences from six species available in GenBank. However, we do not include *Coryneumfoliicola* (CBS 153.32) sequence data in our analyses as its phylogenetic affinities are distant from Coryneaceae (data not shown). Therefore, we use reliable sequences from GenBank to determine the taxonomic placement of our new species.

Based on morphological characters, there are some similarities between *Coryneumheveanum* and related *Coryneum* species, such as acervular conidiomata, fusiform or clavate conidia with pseudosepta (Sutton 1980, Maharachchimbura et al. 2016, Senanayake et al. 2017, 2018). However, *C.heveanum* is distinct from other known taxa including *Coryneumumbonatum* (type species) by conidial measurements, number of pseudosepta and lack of a hyaline tip to the apical cell (Table 2) (Briosi and Farneti 1908, Sutton 1980, Gadgil and Dick 2007, Senanayake et al. 2017, 2018).

Current phylogenetic analyses of combined LSU, ITS and TEF1 alignment are used to clarify the species relationships in *Coryneum* (Figure 1), following Senanayake et al. (2017) and Fan et al. (2018). The phylogenetic tree shows that our species clearly groups with *Coryneum*. In addition, pairwise dissimilarities of DNA sequences of ITS regions between *C.heveanum* and other *Coryneum* species also provide further evidence to justify *C.heveanum* as a new species (Jeewon & Hyde, 2016). Comparison of 599 nucleotides of the ITS nucleotides between *C.heveanum* and *C.umbonatum* (MFLUCC 13-0658 and MFLUCC 15-1110) reveals 90 base pair differences. Comparison of 536 nucleotides of the ITS nucleotides between *C.heveanum* and *C.castaneicola* (43_2) reveals 90 base pair differences. Comparison of 620 nucleotides of the ITS nucleotides between *C.heveanum* and *C.umbonatum* (CBS 199.68) reveals 91 base pair differences. Comparison of 598 nucleotides of the ITS nucleotides between *C.heveanum* and *C.perniciosum* (CBS 130.25) reveals 77 base pair differences. *Coryneumumbonatum* strains (AR3541, MFLUCC 13-0658 and MFLUCC 15-1110) form a distinct lineage, which is in agreement with the results of Fan et al. (2018). However, *Coryneumumbonatum* (CBS 199.68) forms a separate clade with *C.umbonatum* strain AR 3541, MFLUCC 13-0658 and MFLUCC 15-1110 and we cannot verify this taxon based on morphological characters. Previous studies have described the morphological features of *Coryneumumbonatum* but conidial dimensions and number of pseudosepta reported varies significantly from each other (Sutton 1980, Gadgil and Dick 2007, Senanayake et al. 2017, 2018) (Table 2). In addition, some of the *Coryneum* sequences deposited in GenBank (i.e. *C.castaneicola*, *C.depressum*, *C.foliicola*, *C.monodia* and *C.perniciosum*, *C.umbonatum*) lack morphological characteristics and their identities cannot be confirmed. Therefore, these taxa need to be recollected, described and sequenced to determine their taxonomic placement in this family.

## Supplementary Material

XML Treatment for Coryneum heveanum
